# Invariant NKT Cells: Regulation and Function during Viral Infection

**DOI:** 10.1371/journal.ppat.1002838

**Published:** 2012-08-16

**Authors:** Jennifer A. Juno, Yoav Keynan, Keith R. Fowke

**Affiliations:** 1 Department of Medical Microbiology, University of Manitoba, Winnipeg, Manitoba, Canada; 2 Department of Community Health Sciences, University of Manitoba, Winnipeg, Manitoba, Canada; 3 Department of Medical Microbiology, University of Nairobi, Nairobi, Kenya; 4 Department of Internal Medicine, University of Manitoba, Winnipeg, Manitoba, Canada; University of Alberta, Canada

## Abstract

Natural killer T cells (NKT cells) represent a subset of T lymphocytes that express natural killer (NK) cell surface markers. A subset of NKT cells, termed invariant NKT cells (iNKT), express a highly restricted T cell receptor (TCR) and respond to CD1d-restricted lipid ligands. iNKT cells are now appreciated to play an important role in linking innate and adaptive immune responses and have been implicated in infectious disease, allergy, asthma, autoimmunity, and tumor surveillance. Advances in iNKT identification and purification have allowed for the detailed study of iNKT activity in both humans and mice during a variety of chronic and acute infections. Comparison of iNKT function between non-pathogenic simian immunodeficiency virus (SIV) infection models and chronic HIV-infected patients implies a role for iNKT activity in controlling immune activation. *In vitro* studies of influenza infection have revealed novel effector functions of iNKT cells including IL-22 production and modulation of myeloid-derived suppressor cells, but *ex vivo* characterization of human iNKT cells during influenza infection are lacking. Similarly, as recent evidence suggests iNKT involvement in dengue virus pathogenesis, iNKT cells may modulate responses to a number of emerging pathogens. This Review will summarize current knowledge of iNKT involvement in responses to viral infections in both human and mouse models and will identify critical gaps in knowledge and opportunities for future study. We will also highlight recent efforts to harness iNKT ligands as vaccine adjuvants capable of improving vaccination-induced cellular immune responses.

## Introduction

The immune response to invading pathogens requires the successful activation of innate immunity, which informs the development of the subsequent adaptive immune response. A small subset of T lymphocytes expressing surface markers characteristic of both T cells and natural killer (NK) cells are now appreciated to form an important link between the innate and adaptive immune responses. These NKT cells can be activated in both antigen-dependent and independent manners and respond with robust Th1 and Th2 cytokine production, allowing them to exhibit remarkable functional plasticity with both pro-inflammatory and immunoregulatory characteristics. NKT cells can be grouped into several subsets (Table 1), but the most commonly described group is the Type 1 or invariant NKT (iNKT) subset, which is the focus of this Review. iNKTs are highly conserved among mouse, non-human primate (NHP) species, and humans [1]–[4] and are so named due to the expression of a highly restricted T cell receptor (TCR) repertoire. In humans and NHPs, iNKT cells are characterized by expression of a TCR comprised of Vα24-Jα18 paired with Vβ11 (reviewed in Porcelli [5]), while mouse iNKTs express Vα14-Jα18 paired with one of Vβ8.2, Vβ7, or Vβ2 [6]. The majority of iNKTs express CD161 (NK1.1 in mice) and all respond to lipid ligands through CD1d restriction. Despite the low frequency of the iNKT population in the periphery (0.01%–1% of CD3+ lymphocytes in humans), iNKT activity is now appreciated to play important roles in infectious disease, allergy, autoimmunity, and tumor surveillance. This review will focus on the current understanding and gaps in knowledge regarding iNKT function during human viral infection. A description of iNKT function during viral infection in mouse models has previously been reviewed by Diana et al. [7].

**Table 1:** Human and mouse CD1d-restricted NKT cell subsets [130]–[134].

NKT Cell Subset		Mouse	Human
Type I	TCR	Vα14-Jα18; Vβ8.2/7/2	Vα24-Jα18; Vβ11
	Subsets	CD4+, DN	CD4+, CD8+, DN
	Ligand	αGalCer	αGalCer
	Restriction	CD1d	CD1d
	NK receptors	NK1.1+/−	CD161+/−
Type II	TCR	Vα3.2-Jα9 or Vα8; Vβ8	Diverse
	Subsets	CD4+, DN	CD4+, CD8+
	Ligand	Sulfatide, lysosulfatide, lysophosphatidylcholine	Sulfatide, lysosulfatide, lysophosphatidylcholine
	Restriction	CD1d	CD1d
	NK receptors	NK1.1+/−	CD161+

### iNKT Thymic Selection and Development

Current knowledge regarding iNKT thymic selection has recently been thoroughly reviewed by Hu et al. [8]. Like conventional T cells, iNKTs develop in the thymus from CD4+CD8+ thymocytes. Expression of the iNKT TCR is selected by reactivity with CD1d-presented endogenous lipid, which directs cells to the iNKT lineage; the contribution of high-affinity ligand negative selection to iNKT development is still unclear but may also play a role [9], [10]. Signaling from both the TCR and signaling lymphocyte-activation molecule (SLAM) receptors is required for iNKT development. Maturation and proliferation of iNKT cells can occur either in the periphery or the thymus, with mature iNKT cells requiring IL-15 for maintenance [11]. Determinants of iNKT maturation are not fully understood, but were recently shown to involve microRNA-150 expression in mice [12], [13].

### iNKT Activation by Ligand-Dependent and Independent Mechanisms

iNKT TCR–mediated responses are restricted by CD1d, a member of the non-polymorphic CD1 antigen presenting protein family [5], which promotes the presentation of endogenous [14] and pathogen-derived [15]–[26] lipid antigens to the TCR [27]. Although no viral-associated lipid iNKT antigens have been described, iNKT activation in the absence of a pathogen-derived lipid antigen can occur in a CD1d-dependent or independent manner (reviewed in Brigl et al. [22] and Matsuda et al. [28]). iNKT activation by antigen presenting cell (APC)-mediated lipid antigen presentation involves IL-12 production and is strongly dependent on CD40/CD40L interactions [29], with low levels of CD40L being detectible *ex vivo* on the surface of iNKT cells [30], [31] and intracellular, pre-formed CD40L mobilized upon activation [32]. Numerous pathogen-derived lipid antigens have now been identified from bacterial species (reviewed in [33]) and the endogenous lipid β-D-glucopyranosylceramide was recently shown to accumulate in APCs following infection and to activate mouse and human iNKTs [14]. Additionally, both gram negative and gram positive bacteria are capable of activating iNKT cells via TLR stimulation of, and IL-12/IFNα/β production by, APCs [26], [34]–[37]. This mechanism appears to require CD1d-restricted presentation of endogenous lipid. Finally, non-specific CD1d-independent iNKT activation can occur in the context of lipopolysaccharide (LPS)-induced APC production of IL-12 and IL-18 [38]. Given the lack of viral lipid antigens available for CD1d presentation, the capacity to be activated by APC cytokine production allows the iNKT subset to respond to viral infections as well as bacterial and parasitic infections. Indeed, new evidence demonstrates that weak TCR stimulation by endogenous lipids temporarily “primes” iNKT cells to rapidly respond to cytokine activation signals, emphasizing the broad, innate responsiveness of the iNKT subset during infection [39].

### iNKT Subsets and Functional Capacity

**Figure 1:**
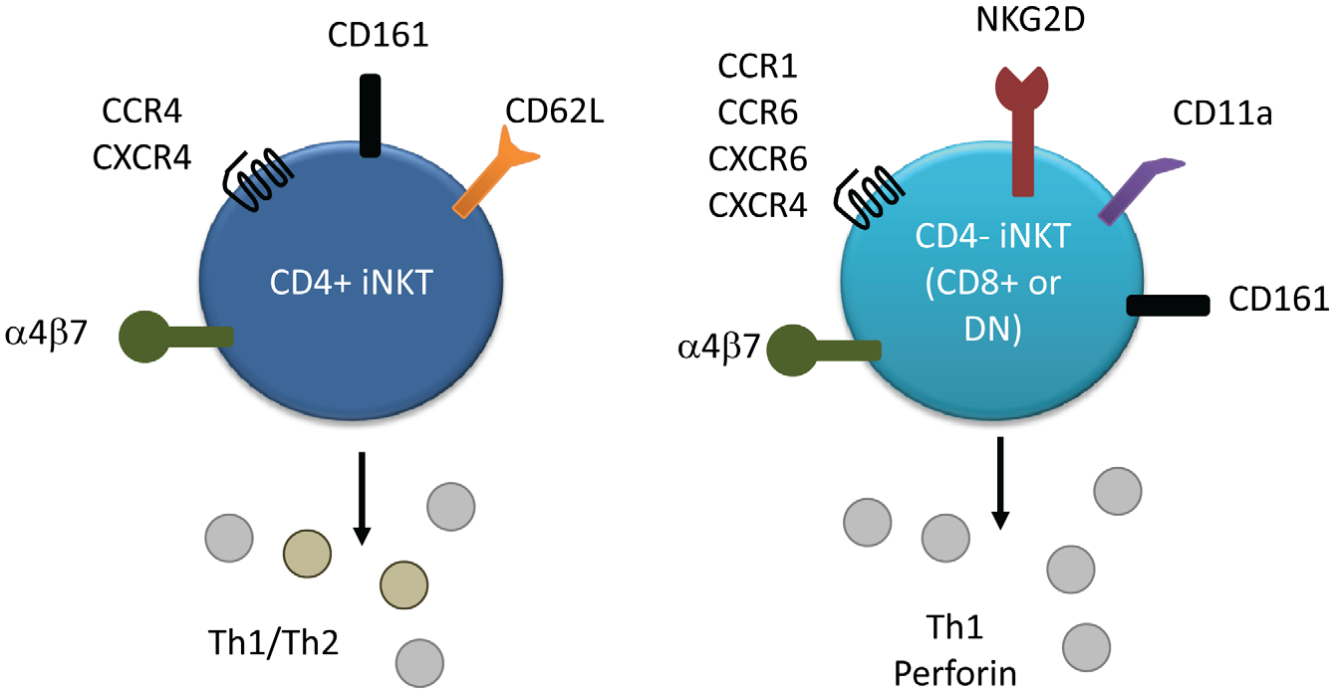
Surface marker and cytokine expression of human iNKT cell subsets. Both subsets express CD161, α4β7, and high levels of CXCR4. CD4+ iNKTs preferentially express CCR4 and CD62L, and produce both Th1 and Th2 cytokines. CD4− iNKTs preferentially express chemokine receptors CCR1, CCR6, and CXCR6, as well as CD11a and NKG2D. This subset secretes predominately Th1 cytokines and more quickly secretes perforin than the CD4+ subset.

Human iNKTs express CD4 and CD8α [40], [41], allowing iNKT subsets to be defined as CD4+, CD4−CD8− (DN), or CD8+. Subset-specific differences in surface marker expression have been described (Figure 1) [30], with CD4+ iNKTs exhibiting lower expression of CCR5 but increased expression of CCR4 compared to the CD4− subset, which characteristically expresses CCR1, CCR6, CXCR6, and NKG2D (reviewed in Kim et al. [42]). All iNKTs express high levels of CXCR3 and CXCR4 and typically exhibit an effector/memory phenotype [43], [44]. The CD4− subset tends to express low levels of CD62L but higher levels of CD11a, suggesting a tissue-infiltrating phenotype, while the CD4+ subset preferentially expresses CD62L and therefore exhibits a lymph node homing phenotype [45]. CD4 and CD8 are both expressed on thymic iNKT cells, but the CD4+ subset predominates. Expansion in the periphery therefore appears to account for the increased proportion of CD8+/DN iNKTs observed outside the thymus [46]. While CD4 expression has known functional consequences during iNKT activation [47], [48], a similar functional impact for CD8 expression has not been described.

**Figure 2:**
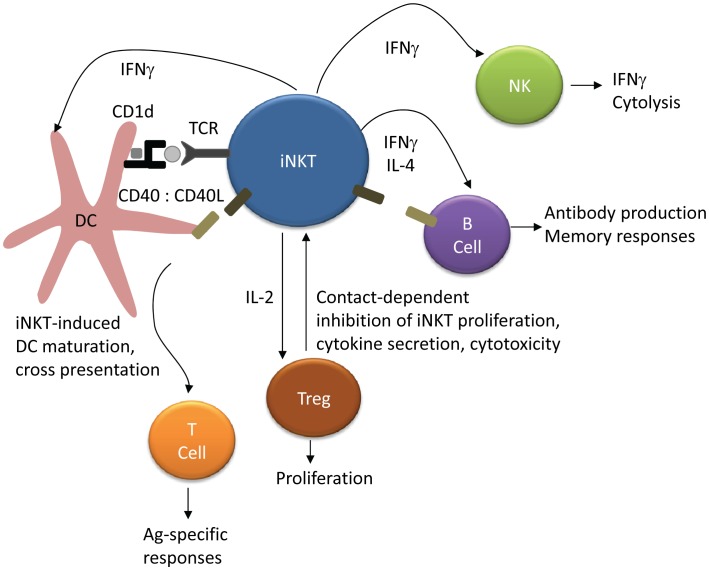
iNKT regulation of NK, T cell, and B cell activation. Presentation of lipid antigen to iNKT cells by DCs leads to iNKT activation and upregulation of CD40L. CD40–CD40L interactions and iNKT cytokine secretion promotes DC activation and maturation, which in turn leads to antigen cross-presentation and augmentation of CD4+ and CD8+ T cell responses. iNKT IFNγ secretion rapidly activates NK cells and induces further IFNγ secretion. iNKTs can substitute for CD4+ T cell help in B cell activation through CD40–CD40L interactions, and iNKT activation improves antibody titres and B cell memory responses. Finally, iNKT production of IL-2 induces regulatory T cell (Treg) proliferation, while Tregs can also inhibit iNKT proliferation and functional responses.

A hallmark of iNKT activation is the rapid production of a vast array of cytokines and chemokines [49], [50] including IFNγ, TNFα, TGFβ, GM-CSF, IL-2, IL-4, IL-5, IL-6, IL-10, IL-13, IL-17, IL-21, RANTES, Eotaxin, MIP-1α, and MIP-1β (reviewed in Matsuda et al. and Tessmer et al. [28], [49]). CD4+ iNKTs produce both Th1 and Th2 cytokines, while CD4− iNKTs generally produce only Th1 cytokines [30], [51] (reviewed in Kim et al. [42]). Other iNKT effector functions include perforin/granzyme release [28], [51], [52], and Fas/FasL-mediated cytotoxicity [28], [49]. iNKTs can play an important role in the activation and regulation of multiple immune cell subsets (Figure 2), including NK, T cell, regulatory T cell, and B cell activation [53]–[55]. Stimulation of iNKT cells in conjunction with soluble T cell antigen enhances both CD4+ and CD8+ antigen-specific responses via a mechanism involving CD40 signaling [56]. Similarly, iNKT activation improves antibody titres, substitutes for CD4+ T cell help to B cells, and enhances B cell memory in mice [57].

The functional plasticity of iNKT cells, combined with their ability to modulate activation of other immune cell subsets, suggests that they may play both a protective role in controlling viral infection and a detrimental role in enhancing viral pathogenesis. Here, we review the current understanding of the roles of iNKT cells in human viral infections, with particular focus on HIV and influenza infection (summarized in Table 2).

**Table 2:** Summary of iNKT studies in viral pathogenesis.

	Mouse		Human	
	Frequency	Function	Frequency	Function
HIV	N/A	N/A	Depletion of total and CD4+ subset [45], [58]; variable recovery after HAART [58], [60], [64]–[66]	Inhibition of IFNγ, IL-4 and proliferation [61], [64], [70]; iNKT cells demonstrate anti-HIV activity [61]
HBV	Increase in hepatic type II NKT cells during acute hepatitis [135]	Activation enhances HBV-specific T cell responses [87]; promote IFNγ-dependent viral inhibition [85]	N/A	N/A
HCV	N/A	N/A	Variable depletion following infection in viremic individuals [91]–[94]	CXCR3 upregulation [94]; greater Th2 cytokine production after expansion [94]
HSV	N/A	Required for viral load control, protection from mortality [105], [106]	N/A	CD1d downregulation reduces iNKT activation [102]
Influenza	N/A	Activation promotes effective NK and CD8+ response [113]; control of viral titre [111]	N/A	Activation reduces the suppressive capacity of MDSCs, improves antigen-specific responses [110]

HAART, highly active antiretroviral therapy; MDSC, myeloid-derived suppressor cell.

## Chronic Viral Infections

### Human Immunodeficiency Virus

#### CD4+ iNKT depletion

iNKT cell frequency is significantly reduced among HIV-1 positive individuals [45], [58], with a specific depletion of the CD4+ iNKT subset compared to the CD4− subset [45]. Longitudinal analysis of pre-seroconversion and 1 year and 5 year post-seroconversion samples demonstrated significant iNKT loss within the first year of infection, with continual declines by 5 years [58]. Expression of CCR5 and CXCR4 on CD4+ iNKTs [43], [45], [59] makes them susceptible to infection with R5-tropic, X4-tropic, and primary isolate viruses [43], [45], [59], resulting in the preferential depletion of CD4+ iNKT cells during *in vitro* infection [45]. The lack of change in CD4− iNKT populations during *in vitro* infection suggests that loss of the CD4+ subset is not largely due to CD4 downregulation. Replication of similar studies in a number of cohorts has largely confirmed these initial observations [60]–[63], although the impact of highly active antiretroviral therapy (HAART) on iNKT cell reconstitution remains controversial [58], [64]–[66] and the kinetics of iNKT reconstitution appear to be slower than that of conventional CD4+ T cells [62], [67].

Although it is now agreed that iNKT cells, particularly CD4+ iNKTs, are depleted during HIV-1 infection, less data is available to clarify the impact of this depletion on disease progression and viral pathogenesis. While it appears that the iNKT subset is involved in the host response to viral infection, it is unknown whether iNKT activation could control HIV replication and immune activation, or what role the iNKT subset might play in anti-tumor responses and prevention of opportunistic infections in immunocompromised hosts [68]. One study to date has demonstrated iNKT cell culture supernatant inhibition of HIV p24 production during *in vitro* CD4+ T cell infection, which was shown to be IFNγ-dependent [61]. In a study of risk factors involved in developing cancer among HIV-1 positive women, NKT cell frequency was associated with a reduced risk of cancer [69]. While further studies are required to assess the increased risk of progression or co-infection, if any, associated with iNKT decline, it is clear that both iNKT number and function are affected by HIV infection, as discussed below.

#### iNKT dysfunction

Even among individuals with minimal iNKT depletion during HIV-1 infection, the iNKT subset displays functional impairment [61], [64], [70]. Both CD4+ and CD4− iNKTs exhibit reduced proliferation and IFNγ, TNFα, and IL-4 secretion in response to αGalCer/IL-2/PMA stimulation [61], [64], [70], with variable restoration among HAART recipients. Increased iNKT expression of exhaustion marker programmed death (PD)-1 was reported among HIV-1 positive individuals in one study [64], but PD-1 levels did not significantly correlate with IFNγ production or proliferative capacity and PD-1 blockade did not restore iNKT function. While Moll et al. suggest that this implies an irreversibly exhausted phenotype, the expression and functional impact of other inhibitory receptors such as 2B4, Tim-3, and LAG-3 on the iNKT subset during HIV-1 infection remains unknown. As evidence now suggests that the function of Tim-3 differs between T cell and NK cell subsets [71], [72], the precise impact of exhaustion marker regulation on iNKT cells during infection must be determined. Additionally, in the Vasan et al. study, stimulations were carried out on iNKT-enriched PBMC cultures that were B cell– and CD8+ T cell–depleted [61]. Given that the unique functional properties of CD8+ iNKT cells and the ability of B cells to present lipid antigen via CD1d are now appreciated, the depletion of these subsets could influence the cytokine production of the iNKT population. More data is also required to address the dysfunction of CD8+ versus DN iNKT subsets during HIV infection, as studies to date have often failed to differentiate these subsets.

#### Non-human primates and SIV infection

Other clues as to the importance of iNKT activation during HIV-1 infection may come from NHP studies of SIV infection. *In vivo* infection of macaques with SHIV_mn229_ and SIV_mac251_ resulted in CD4+ iNKT depletion similar to human HIV-1 infection, and iNKT depletion was tightly correlated to CD4 decline [73]. Animals capable of viral control exhibited reduced CD4+ iNKT decline, and iNKT levels were inversely correlated with viral load. The similarities in iNKT depletion between HIV and SIV infection provide a model to investigate iNKT activation during natural control of infection. Unlike macaques, Sooty mangabeys (SM) control immune activation during chronic SIV infection and do not exhibit progressive immunodeficiency. SM iNKT cells are either CD8+ or CD4−CD8− and express neither CD4 nor CCR5 [74]. As a result, the iNKT subset is maintained following infection and exhibits no impairment in IFNγ production. Given the capacity of SM iNKTs to produce IFNγ, TNFα, IL-2, IL-13, and IL-10 and to degranulate in response to αGalCer stimulation, the authors speculate that iNKTs may play a role in controlling immune activation in this model of natural infection. As murine iNKT IL-4 and IL-10 production can induce regulatory T cell (Treg) development [75], the production of IL-10 by SM iNKTs is of particular interest. Additionally, iNKT-pDC cross-talk during mouse viral infection can induce naïve T cell differentiation into Tregs, suggesting another potential mechanism of iNKT-mediated Treg activation [76]. Maintenance of Tregs during SIV infection is a characteristic of natural SIV control [77], and despite the low frequency of peripheral iNKT cells, the role of iNKT activation in promoting Treg maintenance and controlling immune activation during infection should be further examined [74].

#### CD1d downregulation

While iNKT cells are depleted and exhausted during HIV infection, CD1d expression is also modulated by the virus itself. The HIV-1 protein Nef, responsible for the downregulation of MHC-I A and B alleles [78], also downregulates CD1d via a common tyrosine-based motif [79], [80]. This downregulation was shown *in vitro* to reduce NKT activation and IFNγ secretion after αGalCer stimulation [79], [80].

### Hepatitis

Murine NKT cells are highly enriched in the liver (comprising 10%–30% of T cells) [81], [82], and murine models have clearly demonstrated a crucial role for NKT cell activation in mediating liver pathology in viral- and ConA-induced hepatitis [83]. While many studies have focused on αGalCer-mediated iNKT activation and autoimmune-like models of hepatitis, less is known about the role of NKT and iNKT cells in control of acute and chronic hepatitis B virus (HBV) and hepatitis C virus (HCV) infection in humans. Although human iNKTs do not appear to be highly enriched in the liver compared to peripheral blood, further characterization of human hepatic iNKT subsets is required [84]. Unlike studies of stringently defined iNKT cells in HIV infection, mouse hepatitis studies include a range of NKT subsets and definitions, making it more difficult to draw comparisons from study to study.

#### Hepatitis B virus

Transgenic mouse models of HBV infection have suggested iNKT control of HBV replication through hepatic IFNα/β/γ induction and NK activation [85], [86]. αGalCer-activated Vα14+ iNKT cells also enhance the generation of HBV-specific cytotoxic T lymphocytes (CTLs) following HBsAg-immunization [87], suggesting a potential mechanism by which to promote viral clearance during chronic infection. Studies of NKT function in human HBV infection are currently lacking. Injection of αGalCer in a clinical trial resulted in a significant decrease in iNKT (Vα24+Vβ11+) frequency following treatment, but only one patient exhibited a sustained decrease in HBV DNA levels [88]. Other HBV literature reports only on NKT cells (defined as CD3+CD56+), a cell subset that does not necessarily overlap with the iNKT subset. One group reported a significant drop in NKT (CD3+CD56+) frequency in the first weeks after hospital admission among acute hepatitis B patients, and suggested trafficking of NKT cells to the liver as a potential explanation [89], while a study in India reported significantly increased NKT (CD3+CD56+/CD16+) frequency among acute, but not fulminant, HBV cases [90]. Further characterization of human NKT cells, including more specific delineation of iNKT/NKT subsets, during acute and chronic HBV infection will be required to understand their role in innate immune control of the virus.

#### Hepatitis C virus

Description of peripheral and hepatic iNKT cells during human HCV infection has been highly inconsistent. One study reported significantly lower peripheral blood iNKT (Vα24+Vβ11+) frequency among viremic compared to aviremic HCV seropositive individuals and healthy controls [91]. A similar depletion of hepatic Vα24+ iNKTs was observed among cirrhotic HCV disease patients [92]. Conversely, other studies reported no change in peripheral iNKT frequency between healthy and seropositive individuals [93], [94], nor any correlation with serum HCV RNA titre [94]. Longitudinal analysis showed no change in iNKT frequency following antiviral therapy, or differences between responders and nonresponders [93].

Functional data suggests that iNKT cells may traffic to the liver during HCV infection and acquire a fibrogenic cytokine producing profile. CXCR3 is upregulated on iNKT cells among HCV+ patients [94], possibly due to the increased hepatic levels of IP-10 and MIG during infection [95], [96]. Following expansion of iNKTs derived from HCV+ individuals, IFNγ production negatively correlated and IL-4 positively correlated with serum RNA titre, indicating a potential role for iNKTs in control of HCV replication. Interestingly, iNKTs from HCV+ patients produced more IL-13 and tended toward greater Th2 cytokine production [94]. Given that iNKT cells contribute to liver fibrosis during chronic viral hepatitis via production of IL-4 and IL-13 [97]–[100], this data supports the idea of iNKT functional modification toward a pathogenic cytokine secretion profile.

#### Latent/relapsing viruses

*HSV*. In the context of herpes virus infections, evidence is emerging to support viral interference of iNKT function. Kaposi's sarcoma-associated herpesvirus (KSHV) was the first to be shown to possess the ability to downregulate CD1d expression, an effect mediated by the viral modulation of immune recognition proteins MIR1 and MIR2 [101]. Similarly, herpes simplex virus type I (HSV-1) infection of human peripheral monocytes and immature dendritic cells results in rapid downregulation of CD1d expression via glycoprotein B and US3 [102], [103]. This downregulation results in decreased DC-mediated activation of human NKT cell lines and is thought to facilitate viral evasion of the iNKT-mediated immune response. Interestingly, HSV infection of keratinoctyes does not induce CD1d downregulation, but, through a contact-dependent mechanism, inhibits iNKT cytokine secretion and induces an anergic-like iNKT phenotype [104]. The mechanism of inhibition was not determined, but was not mediated by iNKT PD-1 or Tim-3 expression, suggesting the involvement of an additional, dominant inhibitory pathway. Given the lack of effect of PD-1 or Tim-3 blocking in restoring iNKT function during viral infection (HSV and HIV), it remains to be seen whether multiple inhibitory receptors contribute in each case. The effect of NKT cells in the early response to infection has been studied using a zosteriform model of HSV-1 infection. iNKT-deficient mice have been shown to suffer increased morbidity, enhanced spread of the virus in the nervous system, and diminished ability to clear the virus [105].

In murine models, CD1d^−/−^ mice exhibit significantly higher HSV-1 viral load within dorsal root ganglia, larger skin lesions, and greater neuronal death than wild-type mice, indicating that efficient early viral control requires intact iNKT cells [106]. These results could not, however, be replicated by another group using a different viral strain [107], although the differences in virulence between the viruses used in these studies is worth noting [108]. Similarly, susceptibility of mice to intravaginal challenge with HSV-2 has been studied in several naïve knockout mouse strains. The NKT-deficient mice exhibited intermediate mortality and a 10-fold lower lethal dose compared to wild-type mice [109].

## Acute Viral Infection

### Influenza

#### iNKT cells in host response to influenza infection

**Figure 3:**
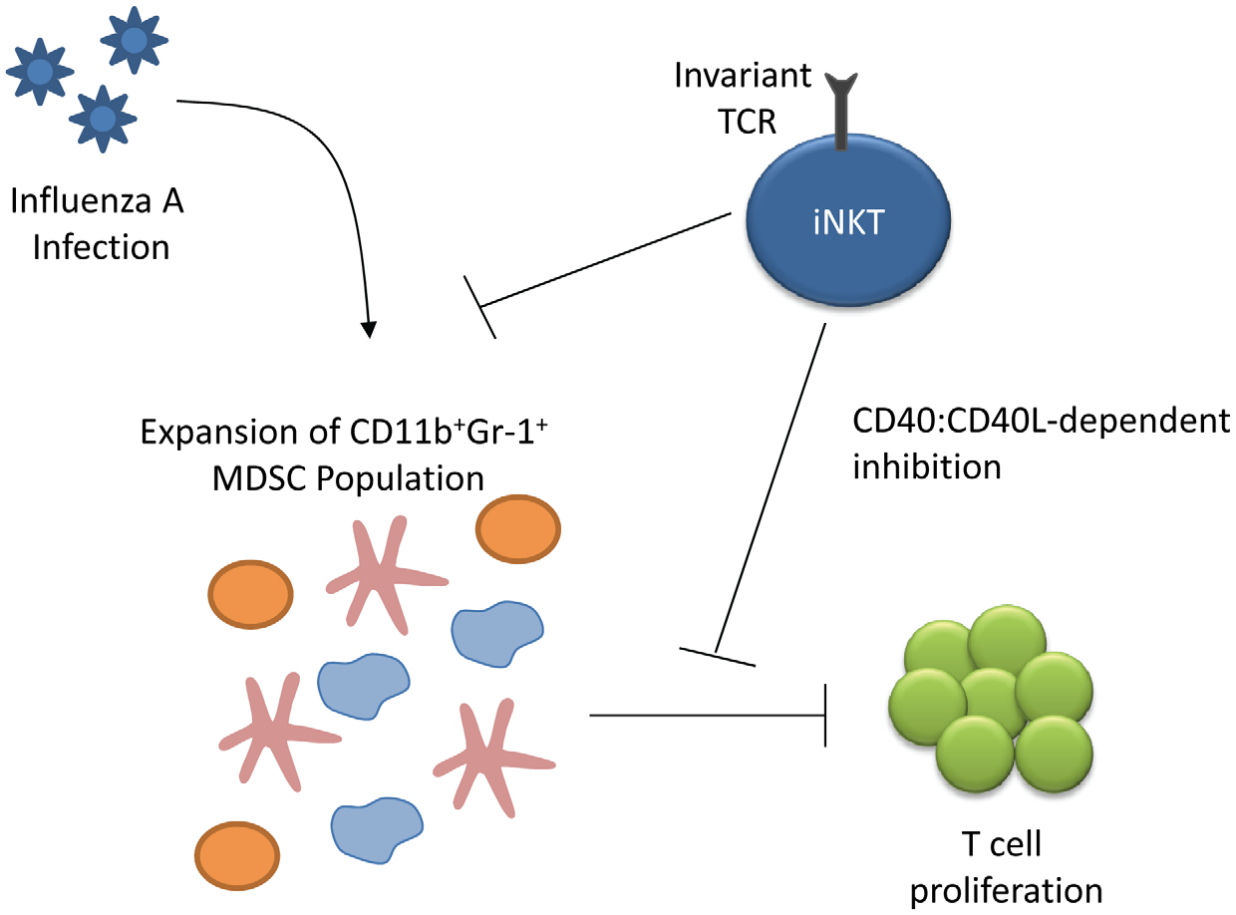
iNKT modulation of myeloid-derived suppressor cells (MDSCs) elicited during influenza A infection. Influenza infection leads to the expansion of the MDSC population (comprised of immature dendritic cells, immature macrophages, and granulocytes), which can inhibit T cell proliferation *in vivo* and *in vitro*. iNKT cells suppress both the expansion of the MDSCs and the suppressive effect of MDSCs in a CD40-CD40L-dependent manner [110].

A novel role for iNKT cells in modulating immune activity was discovered when De Santo et al. reported the identification of myeloid-derived suppressor cells (MDSCs) that could inhibit influenza-specific immune responses and result in increased viral titres and mortality [110]. The group demonstrated that in both mice and humans, iNKT cells functioned to reduce the suppressive capacity of the MDSCs and improved influenza-specific responses (Figure 3). Similarly, activation of iNKT cells boosted early innate immune responses and reduced viral titre [111]. Although iNKTs have not previously been reported to produce IL-22, Paget et al. recently reported that activation of DC TLR7 and RIG-I during murine H3N2 infection results in IL-1β- and IL-23-mediated signals that induce iNKT IL-22 secretion [112]. While IL-22 production was not found to affect viral replication, it did protect epithelial cells from damage *in vitro*. Influenza infection of CD1d-deficient mice also suggests that iNKT-mediated IFNγ production is required for full NK and CD8+ T cell activation and antiviral activity [113], although these results are inconsistent with other studies of CD1^−/−^ mice [114]. In a high pathogenicity model of murine influenza infection, iNKT cells were implicated in the control of infiltrating inflammatory monocytes. Activated iNKT cells were also shown to directly lyse infected monocytes *in vitro* [115]. Increased consistency in the virulence of strains used in challenge experiments and the genetic background of mouse strains will be required in order to conclusively determine the effects of iNKT activation during influenza infection. To our knowledge, only one study has examined iNKT frequency during human influenza infection, but did report a 20% decrease in absolute NKT counts among severe cases of pandemic H1N1 infection [116].

#### Use of αGalCer as a vaccine adjuvant to activate iNKT cells

The vast majority of literature on iNKT cells in influenza infection focuses on the role of αGalCer and iNKT activation as a vaccine adjuvant in mouse models. Initially, it was shown that nasal administration of αGalCer with the antigen PR8 HA (influenza virus A/PR/8/34 (PR8, H1N1)) induced high levels of systemic IgG and mucosal S-IgA Abs, high levels of IFN-γ and IL-4 both locally and systemically, and Ag-specific CTLs. These responses were associated with complete protection against an influenza viral challenge [117]. Subsequent studies documented augmented influenza antibody responses induced by co-administration of vaccine with αGalCer [57], [118]. The study by Galli et al. reported an increased influenza-specific CD4^+^ T cell response after co-administration of vaccine with the adjuvant. They demonstrated that the adjuvant led to activation of iNKT cells, which in turn resulted in antibody responses even in the absence of CD4+ T cells (MHC class II knockout mice), an effect not reproduced by T cell adjuvants such as alum. The authors concluded that iNKT cells can compensate for the absence of CD4+ T cell help [57].

Several lines of evidence also suggest that the stimulation of iNKT cells influences the subsequent cell mediated response to influenza. Administration of αGalCer with a high dose of an inactivated, non-replicating virus had a strong iNKT activating effect; however, this was accompanied by diminished peak CD8+ response to the immunodominant nucleoprotein epitope (NP_366_). Interestingly, increased NP_366_-specific memory CD8+ responses were demonstrated after 6 weeks in the group that received the adjuvant. Taken together, this study indicates a blunted antigen-specific effector CTL response that is followed by an enhanced CD8+ recall [119]. Similarly, injection of αGalCer during murine cytomegalovirus infection also resulted in increased CD8+ central memory cell frequency, further supporting a role for iNKT activation in antigen-specific memory responses [120].

The potential for boosting of both antibody responses and CD8+ memory by stimulation of iNKT cells is appealing in the context of providing cross-protection against emerging strains of influenza. Use of αGalCer as an adjuvant for a live attenuated NS1truncated vaccine has been shown to increase IgG, IgG1, and IgG2a antibodies as well as IFN-γ secreting CD8+ T cells, in an iNKT-dependent manner [121]. Indeed, cross-protection induced by mucosal influenza vaccine along with iNKT cell adjuvant was illustrated by high levels of nasal IgA and cross-protection against a challenge with a non-vaccine strain [122]. Similarly, Lee et al. used two αGalCer analogues with different cytokine release profiles along with inactivated influenza vaccine and were able to induce antibody responses and achieve better cellular immune responses; however, the ability to induce cross-protection was not directly studied [123]. Overall, the use of αGalCer as a vaccine adjuvant to stimulate iNKT cell activation may result in an enhanced mucosal antibody response, improved generation of CD8+ memory, and greater responses to recall antigen. αGalCer may be a particularly useful adjuvant for mucosal immunizations, as mucosal iNKT cells do not become anergic following activation, in contrast to some cases of peripheral iNKT activation [124]. Biochemical modifications of CD1d ligands to produce αGalCer analogues that elicit specific iNKT cytokine secretion profiles will further enhance the utility of iNKT activation as immunotherapy [125], [126]. The fine-tuning of this technique to induce robust memory and cross-protection against emerging influenza strains is promising, and provides a new avenue for vaccine research.

## Conclusions and Future Directions

Since the identification of iNKT cells just over a decade ago, better characterization of CD4+ and CD8+ subsets and description of the growing list of roles they play in bridging innate and adaptive responses has led to appreciation of their importance in the orchestrated response to viral infections (summarized in Table 2). Perhaps most impressive is the amount of information that has been collected in the HIV field with ample evidence of the targeting of these cells by the virus and specific viral effects on CD1d expression, leading to early depletion and dysfunction of the iNKT population. Many questions remain, however, with regards to the kinetics of these changes immediately after acquisition of HIV and the true potential of antiretroviral therapy to reverse dysfunction. NHP studies may play an important role in illuminating whether iNKT cells can contribute to protection from infection at mucosal surfaces or to the control of immune activation and disease progression. Determining whether iNKT cells play a similar role in chronic HBV and HCV infections will require a focus on studies of human infection and improved consistency in the detection and definition of iNKT populations.

In contrast to the plethora of research in the context of HIV as well as other chronic and persistent infections, a paucity of data is available in the context of acute, resolving infections. The vast majority of studies are based on murine models with obvious limitations in their applicability to humans. An accumulation of excellent studies focused on the ability of adjuvants directed at activation of iNKT cells, and co-administered with influenza vaccine formulations, to lead to the generation of a robust humoral and cell mediated immunity is intriguing. Most of these studies use mouse models but hold promise by demonstrating a mechanism that may improve influenza vaccine's ability to result in long lasting CD8+ memory and potentially lead to better cross-protection against newly arising viral strains. As an appreciation of the impact of iNKT activity on viral immunity continues to increase, iNKT cells will likely be found to contribute to host defence in a number of other viral infections. CD1d downregulation appears to be a common immune evasion tactic among viruses, and has also been identified in human papillomavirus (HPV) infection [127]. Some evidence suggests the mast cell–mediated recruitment of NKT cells to sites of dengue virus infection [128] and a potentially detrimental role during pathogenesis in mouse models of infection [129]. As we better understand the mechanisms by which iNKT cells contribute to viral immunity, the therapeutic potential of modulating their activation and function will drive new research avenues.
